# Drawn and Undrawn Poultry

**Published:** 1907-04

**Authors:** 


### EDITORIAL NOTES AND COMMENTS.

The Business Office of the JOURNAL-RECORD is 1007-1008 Century Building.

The Editorial Office is 318-319-320 The Prudential.

Address all Business Commucications to Dr. M. B. Hutchins, Manager.

Make remittances payable to THE ATLANTA JOURNAI-RECORD OF MEDICINE.

On matters pertaining to the Editorial and Original communications, address Dr, Bernard Wolff, Atlanta.

Reprints of original articles will be furnished at cost price. Requests for the same should always be made on THE MANUSCRIPT.

We will present, postpaid, on reqnest, to each contributor of an original article, twenty 20) marked copies of THE JOURNAL-RECORD containing such article.

### DRAWN AND UNDRAWN POULTRY.

We have had occasion before to animadvert upon the subject of drawn and undrawn poultry in respect to their relative preservability and shall now add a word as the matter has lately achieved considerable local newspaper notoriety.

The Board of Health of the city of Atlanta some years ago framed an ordinance prohibiting the sale of undrawn poultry but the ordinance was so vigorously opposed by hotel and restaurant keepers and commission men that it was deemed expedient to withdraw it. This ordinance has been recently revived and is now before the ordinance committee of the City Council for final action. The hotel and restaurant men are again to the front in opposition to the measure and make the contention that undrawn fowls 'keep' better than those from which the entrails have been removed. The opponents to the ordinance endeavor to fortify their cause with an alleged report from some scientists in Washington, the purport of which is that drawn fowls decompose more rapidly than undrawn and that therefore the latter method is preferable. The report may be genuine but from its phraseology and generally light treatment of the subject, to a man from the backwoods, it has the look of the work of a sundowner who secured the fowls from an ashcan. The report parallels in scientific interest the earlier expert testimony in the Thaw case.

In point of fact, this is scarcely an occasion for the application of science, for the use of common sense with some gustatory and olfactory assistance will easily put science to the blink.

Every one knows who has ever cleaned a chicken or witnessed the process as executed by the hands of the presiding genius of the kitchen is aware that a large portion of the alimentary canal is merely a container for highly offensive fecal matter. A fowl's miscellaneous diet lends a combination of malodorous constituents to its excrement which yield a peculiarly nauseous exhalation, as any barefoot boy who has strayed in the barnyard can testify if testimony is needed. This thin filth in undrawn cold-storage poultry is allowed to remain in the body for an indefinite period until by a slow diffusion it has percolated through the tissues of the fowl and thoroughly permeated them with its noxious qualities. The fowl is rendered unwholesome for food and of a taste which may be relished by some people but which is, for the most part, as Mr. Sam Weller remarks very affectin to one's feelins.

But vitiation of taste is by no means so serious an objection as is the absorption of the products of putrefaction when taken into the body and which is often followed by alarming symptoms frequently leading to a fatal termination. This comes insidiously as there is no peculiarity of taste in eating the fowl, thrown out as a warning.

It is a curious anomaly that the contention is made of superior preservability in the undrawn state of no animal used for food except fowls. Why not apply the same arguments to sheep, hogs and beef cattle? Is there any reason why a fowl from which the entrails have not been removed should resist decomposition better than other animals similarly treated?

Now on the other hand, if the viscera be removed, at least one source of putrefactive contamination is eliminated and putrefaction becomes so apparent when occurring that no higher court of appeal than the nose is demanded. The entrance of dry cold air into the abdominal and thoracic cavities can not produce decomposition any more inside than outside and on the contrary can retard indefinitely such changes. In the general movement to secure unequivocally wholesome food for the people it is confidently expeeted that this well-worked fallacy of the superiority of undrawn poultry based upon selfish and mercenary considerations only, will be finally exposed and settled forever.

